# Mental health effects associated with COVID-19 financial assistance in South Korea: a comparison of employment status

**DOI:** 10.1186/s12889-024-18283-6

**Published:** 2024-03-14

**Authors:** Yoonjoo Choi, Eun-Joo Park, Soon-Young Lee, Hee-Yeon Kim, Weon-Young Lee

**Affiliations:** 1 Division of Cancer Prevention, National Cancer Control Institute, National Cancer Center, 323 Ilsan-ro, Ilsandong-gu, Goyang, Republic of Korea; 2 Department of Preventive Medicine, College of Medicine, Chung-Ang University, 84, Heuk-seok dong, Dong-jak gu, Seoul, Republic of Korea; 3 Department of Preventive Medicine, College of Medicine, A-Jou University, 206, Worldcup-Ro, Young-tong gu, Suwon, Republic of Korea; 4 Department of Policy Research, Gyeonggi Welfare Foundation, Gyeong-su daero, Jang-an gu, 1150 Suwon, Republic of Korea

**Keywords:** COVID-19, Mental health, Depression, Anxiety, Financial assistance, South Korea

## Abstract

**Background:**

COVID-19 has created tensions across different sectors of the society, but the impact has been unequal. Vulnerable people have been most affected, especially those with insecure employment and who have experienced economic hardships due to unemployment and lost wages. The combination of social change and economic hardships due to the pandemic increases the risk of poor mental health. Some countries have utilized financial assistance to alleviate economic hardships caused by COVID-19, and in South Korea, the central and local governments have implemented COVID-19 financial assistance. This study analysed the impact of financial assistance on mental health associated with working status during the COVID-19 pandemic in South Korea.

**Methods:**

The participants of this study were randomly selected from residents of Gyeonggi-do after being proportionally allocated by resident registration population status. A total of 1,000 adult males and females aged 19 years or older in Gyeonggi-do who received financial assistance from the central and local governments were selected. A retrospective pre–post-study design was applied, and mental health surveys including the Patient Health Questionnaire-9 (PHQ-9), Generalized Anxiety Disorder 7-item scale (GAD-7) were applied.

**Results:**

The results show that depression scores averaged 5.5 and anxiety scores averaged 4.4 before COVID-19 Financial Assistance. It is similar to the national average of 5.1 and 4.5 respectively at that time. After the assistance, depression scores dropped to 4.5, and anxiety scores dropped to 3.2. Before the assistance, depression and anxiety were higher among temporary day labourers with less job security, and they showed the most significant improvement in mental health. For full-time workers, there was no significant change in anxiety or depression after receiving the assistance.

**Conclusions:**

Financial assistance can provide material resources and also positively affect mental health. In particular, it had a greater impact on the relatively vulnerable groups, such as those in unstable employment.

## Key messages

• Those who are particularly socially and economically vulnerable when in dangerous situations, such as the COVID-19 pandemic, have a greater risk of mental health deterioration.

• Public financial assistance for these individuals is essential and can have a direct effect on alleviating economic difficulties and helping them feel more financially stable in difficult economic conditions.

• Public financial assistance can greatly improve an individual’s resilience and capacity to overcome economically difficult situations.

## Background

In late 2019, the Coronavirus disease 2019 (COVID-19) pandemic hit the world. The pandemic, which was predicted to end quickly, was instead prolonged. In addition to the physical illnesses caused by the virus, the pandemic resulted in challenges to society. High-intensity social distancing measures were implemented for infection prevention, and ways of life changed, including the use of self-isolation and telecommuting and the prevalence of non-face-to-face methods.

Those with stable jobs experienced less economic impact from these changes. However, those with insecure labour, especially those with low incomes, the young and minorities, experienced job, income and life crises [1]. According to the Korean Wage Status by Employment Type, irregular workers earn only 78.5% of the wages of regular workers [2] and are more likely to experience low income or poverty due to the uncertain nature of their jobs. Additionally, according to the Organisation for Economic Co-operation and Development’s (OECD) national self-employment statistics, as of 2020, the self-employed accounted for 6.3% of workers in the United States and 14.7% in the European Union, but made up 24.6% of the workforce in South Korea, making it the sixth highest percentage of self-employed workers among OECD countries [3]. Compared to regular full-time workers, the self-employed are under more pressure and are responsible for all economic and health risks [4–7]. Furthermore, some self-employed people were also in the ‘at-particular risk’ group because their operations were restricted, while unstable workers who were engaged in temporary or daily jobs were exposed to unemployment risk [8].

The number of people complaining of stress, depression, and lethargy was also found to increase as economic difficulties overlapped with the ongoing instability, including infection risk. Studies conducted in six European countries illustrated that economic difficulties caused by unemployment, reduced incomes, and shortened working hours adversely affected mental health [9]. While the deepening employment insecurity due to COVID-19 was strongly associated with depression in the United States [10]. Accordingly, this study highlights that financial stress during the COVID-19 period caused depression and that ‘financial COVID-19 assistance’ was required to provide timely help to those affected financially [11].

The International Labor Organization (ILO) has recommended active fiscal policies, such as cash transfers or financial assistance, to support vulnerable populations during disaster situations [12]. In response, many countries have tried a variety of policies to support their citizens experiencing economic hardship after COVID-19 and have implemented assistance programs such as cash transfers or cash equivalent vouchers. Cash assistance is often used as a one-off or short-term disaster response policy because it is quicker to implement and more accessible to disburse than in-kind or other assistance types [13].

During the height of the COVID-19 pandemic, many countries attempted to introduce financial assistance programs to support the livelihood of their citizens suffering from economic difficulties. Hong Kong provided HK$10,000 to nearly 7 million permanent residents aged 18 and over in March 2020 in response to the Hong Kong protests and COVID-19-induced economic downturn [14].

In April 2020, Japan issued 100,000 yen (USD 700) cash payments to approximately 10 million households with documented post-COVID-19 income losses [15, 16]. Spain became the first country in southern Europe to make payments to around 2.3 million nationals aged 23–65 and legal residents of at least one year. These payments were the difference between the individual’s income and a minimum income threshold, based on the number of people in their household. This began in June 2020 [17, 18]. Togo launched a universal cash transfer program for workers in informal jobs [19], and in South Korea, local governments provided aid in a lump sum ranging from USD 100 to USD 1,000, depending on the number of household members. This was administered through special financial assistance in April 2020 and by the central government in May 2020 [20, 21].

Such financial assistance policies can positively impact mental health not only in economic terms but also in terms of health capital [22]. Reducing financial constraints immediately impacts psychological well-being [23]. Therefore, financial support programs can have a positive impact on the mental health of those suffering from stress, depression and lethargy while experiencing job-related crises, reduced incomes, or other life crises [24]. Previous studies have demonstrated that financial support programs help alleviate mental health issues resulting from situations of financial concern [25, 26]. Additionally, a systematic review analysing the relationship between the Financial Relief program and social determinants of health found increased financial stability, reduced stress and anxiety, and improved mental health levels following cash payments [25].

Since financial stress affects mental health, even in COVID-19 situations there have been many discussions about whether financial support, as well as psychological intervention, could help reduce anxiety and depression in the general population [9, 11, 26]. Small business owners, self-employed people, and workers in unstable jobs who had experienced various difficulties due to social distancing and business restriction policies during COVID-19 showed a significant improvement in negative mental health states in some studies [9, 27, 28]. In light of these prior studies, the present research sought to analyse how implementing South Korea’s financial assistance program affected mental health during the COVID-19 pandemic and explore how that effect varied by employment status.

## Methods

### Study Population

**Fig. 1:**
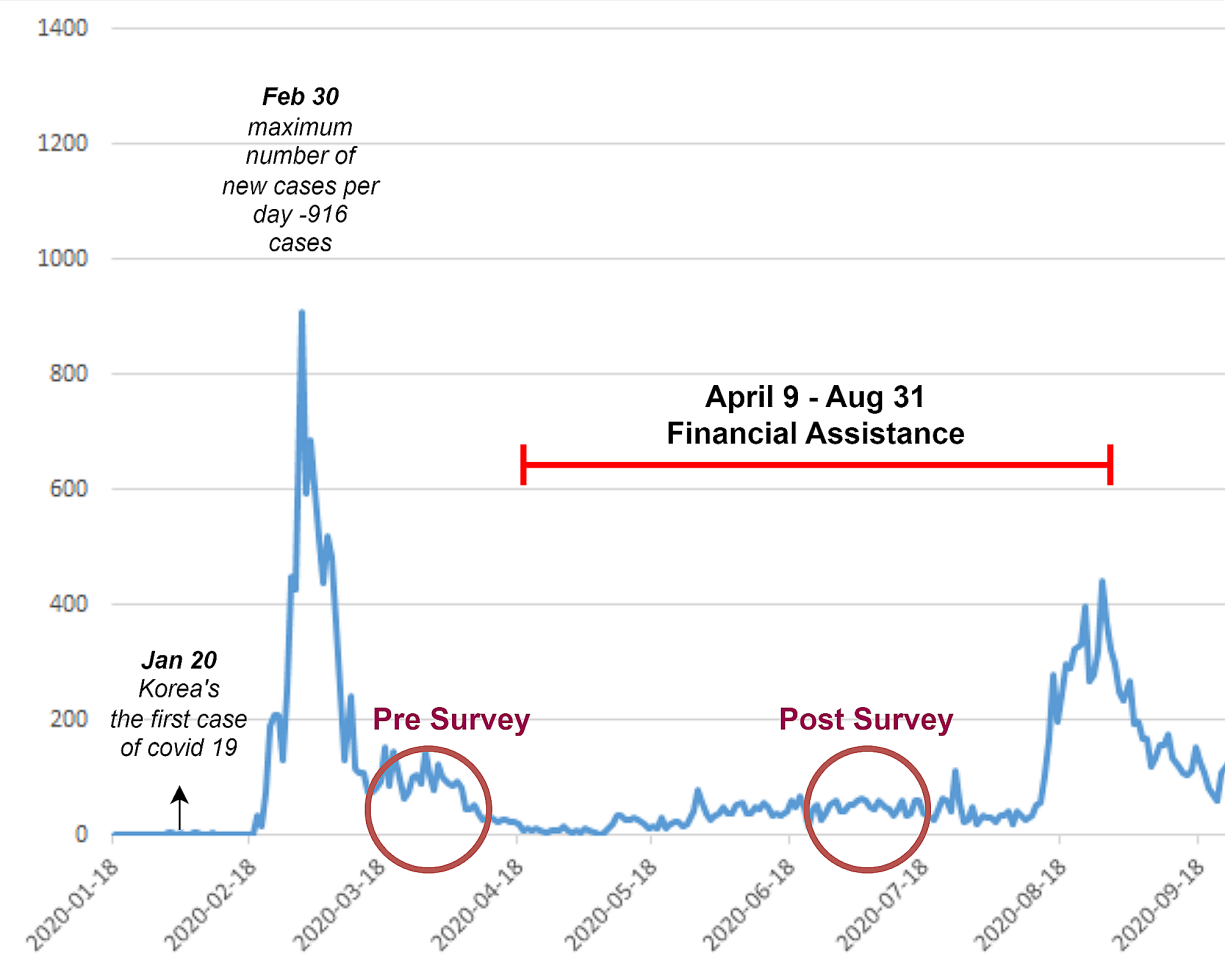
Financial assistance and online survey period

The survey population consisted of an online panel of a specialised survey company. It allocated the sample numbers in proportion to the number of people by gender and age per the Ministry of the Interior and Safety’s resident registration demographics. It then began conducting random sampling. Among them, an online survey was conducted using a structured questionnaire. This involved those who checked the recruitment guide included in the study description and voluntarily participated in the online survey. The survey was conducted until 1,000 people had completed it. This was the determined sample size for the study, and the survey was completed after this sample size was achieved. A total of 9,700 people were invited to participate in the survey over the 11 days of 3–13 July 2020, of which 7,830 (80.7%) did not respond, 414 (4.3%) were excluded due to exceeding the allotted sample size, 233 (2.4%) did not meet the age criteria, and 223 (2.3%) dropped out without completing the survey. The final sample size included 1,000 adult men and women aged 19 and older in Gyeonggi Province. (Fig. 1).

### Study Design

**Fig. 2:**
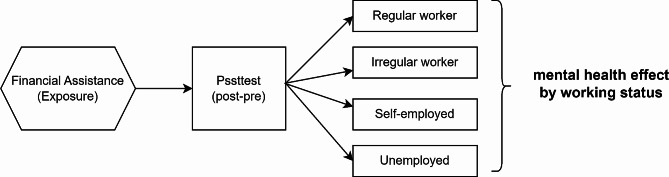
Retrospective post then pre-study design

This study applied a ‘retrospective post then pre-design’ methodology to identify any changes in mental health resulting from receiving COVID-19 financial assistance (Fig. 2). This study was designed to respond to pre-measurement using retrospection of the individual respondent’s previous situation and conducted post-intervention measurements after program intervention [29, 30]. This is a useful research design since it can be used to effectively control and compensate for overestimation errors or response shifts. These can occur when prior measurements are excessively high due to program expectations, education, policies, etc., which are normally involved in traditional pre-post designs [31, 32].

This study had no pre-data associated with emergency COVID-19 financial assistance. In this case, ‘retrospective post then pre-design’ was used to respond, requiring the participants to recall their previous points of view, and allowing their pre-pandemic state of mind to be compared. After sufficiently notifying the respondents about the timing of the retrospective responses, it read, ‘Please respond while recalling around March when the “high-intensity social distancing” was implemented.’

This study was reviewed and approved by the Chung-Ang University IRB (1041078-202009-HRSB-258-01).

## Exposure

In South Korea, the first COVID-19 patient was identified on 20 January 2020, and the number of infected people increased to 916 by the end of February [33]. Social activities began to be reduced due to fears of infection, and there was a sharp decline in offline sales compared to the same month in the previous year [34]. Although there was no lockdown or border controls implemented as in other countries, from 22 May 2020, no more than four people were allowed to gather together, and the number of events, such as weddings, was limited [33]. Thus, both service industry business and manufacturing production fell sharply, causing unemployment to increase [35].

Therefore, to provide income support to people suffering from economic difficulties, the Gyeonggi-do Province unconditionally paid KRW 100,000 (approximately USD 100) per person to all local residents (except non-residents) in April 2020. Single-person households received KRW 100,000, and two-person households received KRW 200,000 in proportion to the number of household members. Additionally, in May 2020, the Korean central government paid disaster support funds to each household in the nation. A sum of KRW 400,000 was paid to single-person households, KRW 600,000 to 2-person households, KRW 800,000 to 3-person households, and KRW 1,000,000 to ≥ 4-person households, in the form of a reduction to their credit card bills, which could be used in the residential area.

Thus, Gyeonggi residents received both subsidies from the central government and the metropolitan government. Accordingly, the total amount paid was KRW 500,000 to single-person households, KRW 800,000 to 2-person households, KRW 1,100,000 to 3-person households, KRW 1,400,000 to 4-person households, and KRW 1,500,000 to 5-person households.

### Employment status

The primary variable examined in this study was employment status. Employment status was divided into four categories: regular worker, irregular worker (including temporary day labourers), self-employed, and unemployed. Irregular workers included fixed-term workers and temporary day labourers. Additionally, the self-employed were defined as a state in which a person employs themselves and can hire others, as defined by the ILO.

### Socio-demographic variables

The demographic variables used in the analysis were gender, age, education (‘high school or less’, ‘college’, ‘graduate or higher’), income (bottom 25%, middle 50%, top 25%), and welfare eligibility (‘yes’, ‘no’). A subjective assessment of health status (‘very good’, ‘good’, ‘normal’, ‘bad’, ‘very bad’), which may be related to mental health, was utilised. These variables were used as covariates.

### Instruments

#### PHQ-9

In this study, a Patient Health Questionnaire-9 (PHQ-9) was used to measure depression. This is a tool that can measure depression experienced in the previous two weeks [36]. It utilises a self-reported questionnaire designed to help detect and diagnose mental diseases that are commonly encountered in primary medical institutions. Many studies have shown that it provides better reliability and validity than other similar tools, and it is widely used for diagnosing major depression disorders [36, 37]. A total of 27 points for 9 questions were used as criteria for diagnosing depression. ‘Moderate’ was indicated if a score of 10 or greater was received, and suicidal ideation could be predicted by question 9 [38].

#### GAD-7

The Generalized Anxiety Disorder 7-item scale (GAD-7) was used to measure anxiety. This is a tool that measures anxiety symptoms experienced in the previous two weeks and was developed for use by primary care medical doctors as a tool for evaluating mental disorders [37, 39]. While it is not used for diagnostic purposes, it represents a useful screening tool because it can rapidly identify if an anxiety disorder is present [40]. A total of 21 points from 7 questions can be used as the criteria for screening anxiety disorders. ‘Moderate’ is generally designated by a score of more than 10 [39].

### Statistical analysis

This study was analysed using Stata, version 16 (StataCorp LLC, College Station, TX, USA). Demographic data was analysed using frequencies, and a repeated measures (RM) analysis of variance (ANOVA) was conducted to determine whether there were differences in depression and anxiety levels before and after receiving financial assistance. RM ANOVA is useful for analysing data in which the same object is measured multiple times over a period. Repeatedly measured data are characterised by correlation, which leads to errors when analysed by *t*-test or ANOVA. RM ANOVA can identify small fluctuations, increasing a study’s accuracy [41]. If the results of ANOVA analysis are statistically significant, it can be inferred that there are differences between groups but it does not identify which groups are different. In addition to employment status, essential demographic characteristics that affect mental health, such as depression and anxiety, were used as covariates. Gender, age, education, income, welfare eligibility, and subjective health status were used. Additionally, a post hoc analysis was performed to examine which groups had different values. If statistical significance was indicated by RM ANOVA, a Bonferroni post hoc test was performed. All statistical significance levels were set at *p* <.05.

## Results

### General characteristics

The final study totalled 1,000 participants, consisting of 50.1% male and 49.9% female. Regarding age, 17.4% were 19–29 years old, 17.9% were 30–39 years old, 21.1% were 40–49 years old, 22.8% were 50–59 and 20.8% were 60 + years old. Regarding education, 18.0% had high school or less, 69.5% had college and 12.5% had graduate degrees or higher. Regarding work status, 47.3% were regular workers, 32.7% were unemployed, 12.6% were self-employed, and 7.4% were irregular workers. Income was 57.5% for the middle 50%, 22.7% for the under 25%, and 19.8% for the upper 25%. A total of 2.8% of the welfare-eligible previously received government assistance. Additionally, 6.2% had excellent subjective health, 34.3% were good, 48.5% were normal, 9.9% were bad, and 1.1% were very bad. Details of the study subjects are presented in Table 1.

**Table 1:** General characteristics of study subjects

		n	%
Sex	Male	501	50.1
	Female	499	49.9
Age	19–29	174	17.4
	30–39	179	17.9
	40–49	211	21.1
	50–59	228	22.8
	≤ 60	208	20.8
Education	High school or less	180	18.0
	College	695	69.5
	Graduate or higher	125	12.5
Work status	Regular worker	473	47.3
	Irregular worker	74	7.4
	Self-employed	126	12.6
	Unemployed	327	32.7
Income	Under 25%	227	22.7
	Middle 50%	575	57.5
	Upper 25%	198	19.8
Welfare eligibility	Yes	28	2.8
	No	972	97.2
Subjective health status	Very good	62	6.2
	Good	343	34.3
	Normal	485	48.5
	Bad	99	9.9
	Very bad	11	1.1

#### PHQ-9

**Fig. 3:**
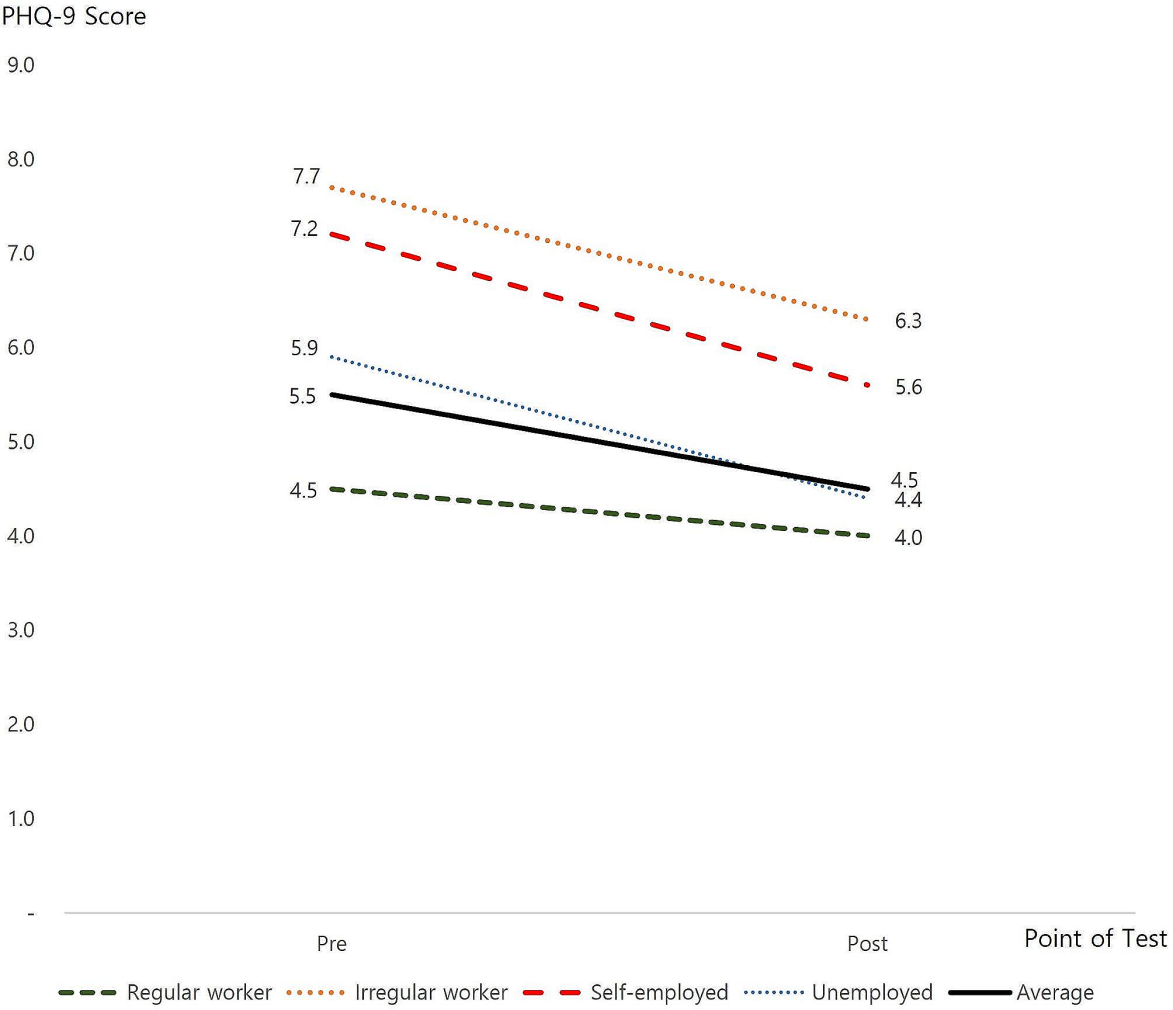
Change in participants’ PHQ-9 scores by employment status

The average PHQ-9 score before the financial assistance was 5.5, similar to the South Korean national average of 5.1 during the same period. It decreased to 4.5 after financial assistance. Analysis of changes in PHQ-9 scores by employment status revealed irregular worker scores before the assistance were 7.7, followed by 7.2 for self-employed workers. Alternatively, regular workers with high job stability produced the lowest scores with 4.5 points. Self-employed workers provided the most considerable change in scores following financial assistance, where a 1.6-point reduction resulted in an average score of 5.6. The score for the unemployed also decreased by 1.5 points, and the score for irregular workers decreased by 1.4 points. However, even after assistance, irregular workers remained the group with the highest score (6.3 points). The before and after PHQ-9 results are shown in Fig. 3.

#### GAD-7

**Fig. 4:**
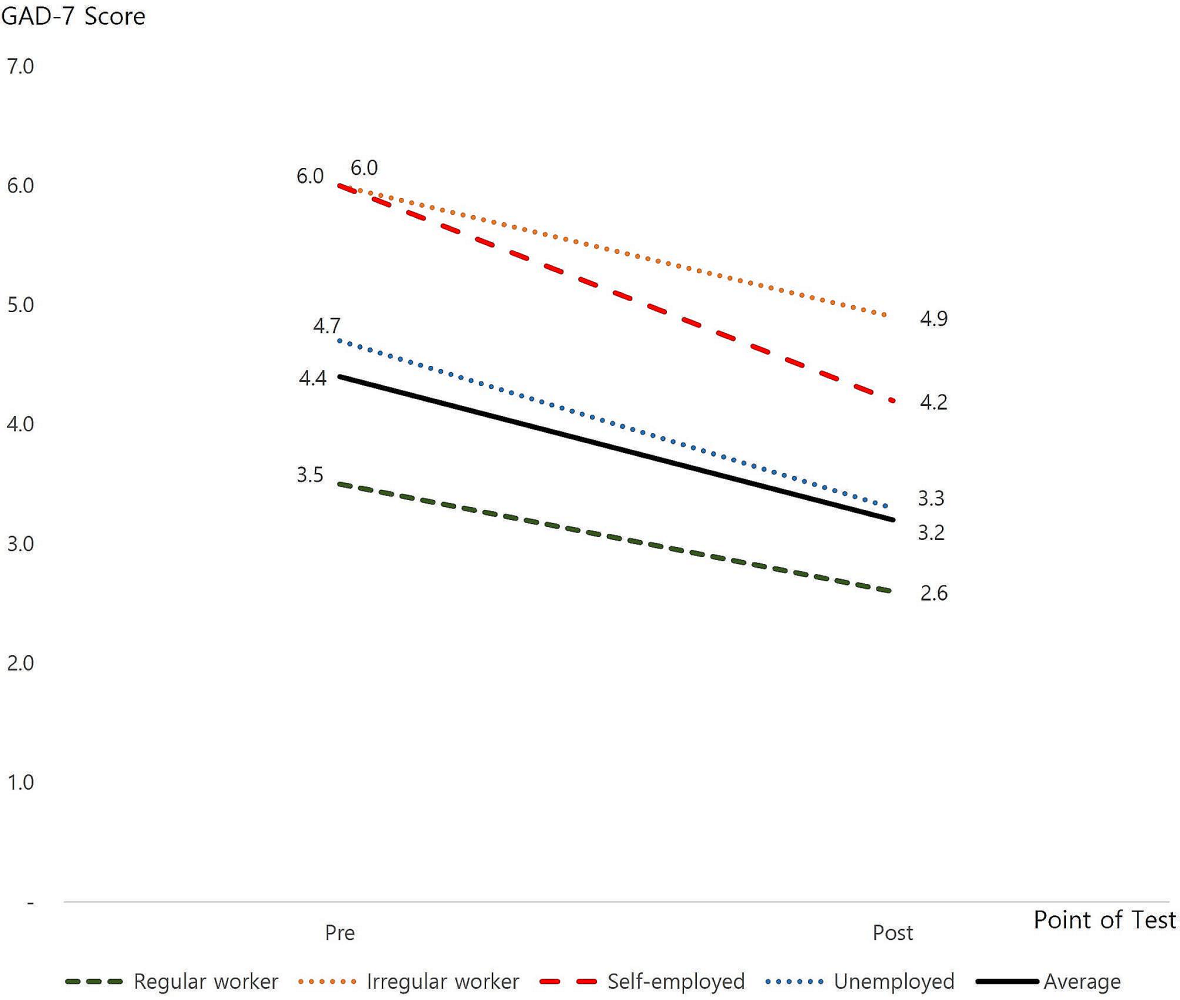
Change in participants’ GAD-7 scores by employment status

The overall average GAD-7 score before COVID-19 financial assistance was 4.4, which was similar to the South Korean National average of 4.5 during the same period. After assistance, it decreased to 3.2. The analysis results indicated a change in GAD-7 scores by employment status, with the scores for irregular workers before the COVID-19 financial assistance, along with irregular and self-employed workers, being the highest at 6.0. Conversely, regular workers with greater job stability scored the lowest with 3.5 points. Again, self-employed workers experienced the most substantial changes following COVID-19 financial assistance, with a reduction of 1.8 points to 4.2. The score for the economically unemployed also decreased by 1.4 points, and the score for irregular workers decreased by 1.1 points yet remained the highest with a score of 4.9, even after payment. The before and after results of the GAD-7 scores are shown in Fig. 4.

#### Repeated measure ANOVA

RM ANOVA was conducted to assess mental health differences according to employment status before and after the COVID-19 financial assistance program was implemented. Here, significant differences were found in the interactions between financial assistance and the employment status groups, as well as both PHQ-9 (*p* =.011) and GAD-7 (*p* =.042) (Table 2).

**Table 2:** RM ANOVA analysis results

		PHQ-9		GAD-7	
Employment status		Pre	Post	Pre	Post
Regular worker		4.5 (5.8)	4.0 (5.6)	3.5 (4.8)	2.6 (4.1)
Irregular worker		7.7 (7.7)	6.3 (6.9)	6.0 (6.5)	4.9 (5.7)
Self-employed		7.2 (7.1)	5.6 (5.9)	6.0 (6.3)	4.2 (5.1)
Unemployed		5.9 (6.7)	4.4 (5.4)	4.7 (5.5)	3.3 (4.6)
source	Group (F/P)	9.745 (0.000)		10.766 (0.000)	
	Time (F/P)	5.134 (0.024)		3.585 (0.059)	
	G×T (F/P)	3.764 (0.011)		2.156 (0.042)	

F: *F*-values in repeated measure ANOVA; P: *p*-value

Gender, age, education, income, welfare eligibility, and subjective health status were used as covariates.

#### Bonferroni post hoc test

Subsequently, a Bonferroni post hoc test was conducted to determine specific differences between employment status groups (Table 3). The PHQ-9 provided the largest difference between regular workers with greater job stability and irregular workers with less employment stability (average difference − 2.498, *p* =.002). Meanwhile, the GAD-7 revealed the greatest difference between regular workers and the self-employed (average difference − 2.147, *p* <.000).

**Table 3:** Results of the Bonferroni test

	Employment status	Average difference	SE	*p*-value	95%CI	
PHQ-9 (ref: Regular worker)	Irregular workers	-2.498^*^	0.700	0.002	-4.349	− 0.647
	Self-employed	-2.388^*^	0.566	0.000	-3.883	− 0.892
	Unemployed	− 0.331	0.415	1.000	-1.427	0.766
GAD-7 (ref: Regular worker)	Irregular workers	-2.124^*^	0.575	0.001	-3.644	− 0.603
	Self-employed	-2.147^*^	0.465	0.000	-3.375	− 0.918
	Unemployed	− 0.526	0.341	0.737	-1.427	0.375

## Discussion

This study analyses the impact of financial assistance on mental health in association with working status during the COVID-19 pandemic in South Korea. A retrospective pre–post-study design was applied, and mental health surveys, such as the PHQ-9 and GAD-7, were applied. Descriptive analysis, RM ANOVA, and Bonferroni post hoc analysis were conducted. The results reveal that before COVID-19 financial assistance, depression scores averaged 5.5 and anxiety scores averaged 4.4. This was similar to the national average of 5.1 and 4.5, respectively, during that period. After the financial assistance, depression scores dropped to 4.5, and anxiety scores dropped to 3.2. Before the assistance, depression and anxiety were higher among temporary day labourers with less job security, among whom the most significant improvement in mental health was observed. For regular workers, there was a slight change in anxiety or depression after receiving financial assistance.

The COVID-19 pandemic affected the global economic recession and prompted a decline in mental health in individuals experiencing financial difficulties, becoming a significant concern. Several studies have shown that employment status and financial problems have a considerable effect on causing depression and anxiety to worsen [10, 11]. However, there has been disagreement regarding whether financial support could help alleviate mental health deterioration [11, 24].

Many studies have analysed the mental health effects of financial assistance programs before COVID-19 [26, 42–44]. However, the COVID-19 period differs from previous periods, and studies have shown that the mental health problems in those who experienced unemployment during the COVID-19 period were more severe than in individuals who had experienced unemployment before the pandemic [45]. Therefore, this study examined how the financial assistance program implemented during the pandemic period affected mental health, such as depression and anxiety, in the general population.

The participants in the study were found to have lower levels of depression and anxiety after the financial assistance compared with before the implementation of the financial assistance. Moreover, we found that depression was highest before the onset of financial assistance in individuals with irregular jobs, followed by those who were self-employed. However, after financial support, the depression score for the self-employed, unemployed, and irregular workers all decreased. Alternatively, for economically stable regular workers, the depression score during the previous period was the lowest, and only slight differences from other employment types were observed after financial assistance.

Likewise, anxiety before the financial assistance was the highest among irregular and self-employed workers, while after financial assistance, the largest reductions were observed in the self-employed, unemployed and irregular workers. Moreover, similar to the scores for depression, regular workers had the lowest anxiety score before financial assistance, and their scores did not change much following assistance.

These findings are consistent with previous studies which have shown that financial assistance programs positively affected social, psychological, and mental health aspects [26, 42–44]. In economically difficult situations, housing, food, medical care, and essential service resources are scarce. In contrast, the introduction of financial assistance programs increases accessibility to these resources. Studies emphasising the positive effects of financial assistance programs revealed that solely alleviating financial difficulties has an immediate impact on psychological well-being [23]. Furthermore, increasing financial stability reduces stress and anxiety, decreases isolation and restores self-esteem [46, 47]. Those who are particularly socially and economically vulnerable in dangerous situations, such as the COVID-19 pandemic, have a greater risk of mental health deterioration [1].

However, it is noteworthy that even after financial assistance, the mental health levels of vulnerable people remain the worst. Financial assistance for them is essential, while assistance can have a direct effect on alleviating economic difficulties and helping them feel more financially stable under challenging economic conditions. Additionally, it can significantly improve capacity and resilience and help overcome economically challenging situations.

This study has certain limitations. The positive mental health changes observed cannot exclude the influence of gradually changing social situations, such as the decrease in confirmed cases before and after the intervention and the relaxation of social distancing intensities. Additionally, this study does not consider the possibility that employment status may have changed before or after the financial assistance program was implemented. Finally, recall bias in responses can exist due to online surveys and the retrospective post-study followed by pre-study design.

However, although there was no existing pre-data, this study attempted to identify the effects of policies in emergencies and compared regular workers with decent mental health levels due to relatively low economic damage alongside self-employed, temporary and unemployed workers. Furthermore, it closely examined the groups that had higher positive mental health changes associated with financial assistance.

## Conclusions

When faced with an unexpected and sudden crisis, socially and economically disadvantaged people are more likely than others to suffer financial hardships and mental health deterioration due to stress. Therefore, it is necessary to provide public financial assistance during disaster situations to not only help alleviate economic hardships but also to build resilience to overcome difficult situations through psychological stability.

## Data Availability

The datasets are not publicly available as they contain information that could compromise the research participant’s confidentiality. If you have any questions about the data or materials in this study, please contact Yoonjoo Choi or the corresponding author.
